# Supplementary material for: Deep Learning and Machine Learning Modeling Identifies Thidiazuron as a Key Modulator of Somatic Embryogenesis and Shoot Organogenesis in Ferula assa-foetida L
Source: Biology (Basel). 2025 Nov 29;14(12):1703. doi: 10.3390/biology14121703 (PMC12730490; doi:10.3390/biology14121703)
Supplement: Supplementary file 1 [file biology-14-01703-s001.zip › Supplementary Tables S1-S4.pdf]

**Supplementary Table S1: CNN resolution computational details**

| Model | Resolution | Accuracy % | Training time (sec) | Total parameters |
|---|---|---|---|---|
| **CNN** | **32x32** | **87** | **9.37** | **159944** |
| **CNN** | **64x64** | **79** | **39.26** | **684232** |
| **CNN** | **128x128** | **78** | **140.67** | **3305672** |

**Supplementary Table S2: CNN augmentation computational details**

| Model | **Resolution** | Approach | **Accuracy %** |
|---|---|---|---|
| CNN | **32x32** | Baseline ( No augmentation) | 87 |
| CNN | **32x32** | With augmentation | 13 |

**Supplementary Table S3: classification accuracy (%) obtained for each fold during 5-fold cross-validation using the same CNN architecture and hyper-parameters**

| Fold | Accuracy % |
|---|---|
| 1 | 81 |
| 2 | 74 |
| 2 | 76 |
| 4 | 86 |
| 5 | 84 |

**Supplementary Table S4: Computational Environment details**

| | |
|---|---|
| **Python version** | **3.13.7** |
| **TensorFlow version** | **2.20.0** |
| **OS** | **Windows** |
| **OS Release** | **11** |
| **Processor** | **Intel64 Family 6 Model 186 Stepping 3, GenuineIntel** |
| **Machine** | **AMD64** |
| **GPU Devices** | **NA** |
