# Supplementary figures and images for: Deep Learning and Machine Learning Modeling Identifies Thidiazuron as a Key Modulator of Somatic Embryogenesis and Shoot Organogenesis in Ferula assa-foetida L
Source: Biology (Basel). 2025 Nov 29;14(12):1703. doi: 10.3390/biology14121703 (PMC12730490; doi:10.3390/biology14121703)

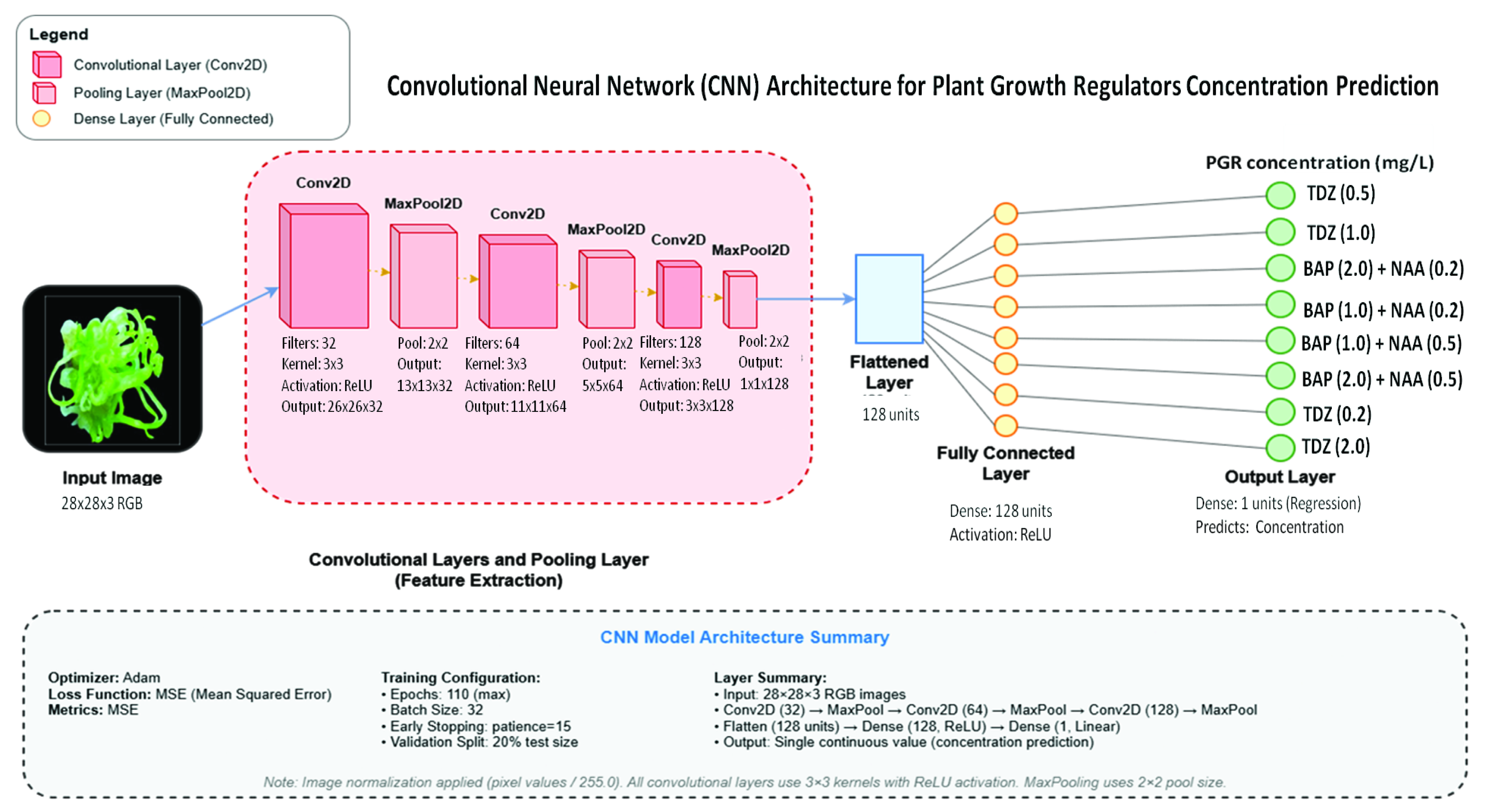

Supplement: Supplementary file 1 [file biology-14-01703-s001.zip › supplementary figure S1.tif]
